# Sex Distribution of Study Samples Reported in American Society of Biomechanics Annual Meeting Abstracts

**DOI:** 10.1371/journal.pone.0118797

**Published:** 2015-03-04

**Authors:** Sarah Bach, Melissa M. Morrow, Kristin D. Zhao, Richard E. Hughes

**Affiliations:** 1 Department of Industrial & Operations Engineering, University of Michigan, Ann Arbor, Michigan, United States of America; 2 Division of Orthopedic Research, Mayo Clinic, Rochester, Minnesota, United States of America; 3 Rehabilitation Medicine Research Center, Physical Medicine and Rehabilitation, Mayo Clinic, Rochester, Minnesota, United States of America; 4 Department of Orthopaedic Surgery, University of Michigan, Ann Arbor, Michigan, United States of America; Ohio State University, UNITED STATES

## Abstract

**Background:**

Study samples should be appropriately selected to maximize generalizability of results. Excluding one sex from studies of conditions that affect both sexes is problematic and has received attention as a public policy issue in the United States, resulting in legislation and recommendations made by the National Institutes of Health to address this deficiency of study designs. It is unknown to what extent biomechanical studies have inappropriately excluded one sex. The objective of this study was to provide objective data on this question.

**Methods:**

A retrospective review of random samples of abstracts presented at American Society of Biomechanics annual meetings from 1983 to 2013 was conducted to assess reporting of sex of study samples and whether the study samples were approximately balanced with respect to sex.

**Findings:**

We did not find a statistically significant increasing trend in the percentage of abstracts reporting sex over time. However, increasing trends were noted in the percentage of abstracts including both sexes (*p* < 0.05) and percentage of abstracts having an “approximately balanced” study sample containing 50 ± 20% females (*p* > 0.05). In 2013 the percentage of abstracts reporting studies having approximately balanced study samples was only 28%, far from the ideal level of 100%.

**Interpretation:**

While there has been modest change since 1983, there remains significant room for improvement in the reporting and composition of experimental studies reported at American Society of Biomechanics annual meetings.

## Introduction

Due to disparities in subject recruitment, the National Institutes of Health (NIH) Revitalization Act of 1993 required the NIH to develop policies regarding inclusion of women and minorities in studies involving human participants. In fact, Title 42 U.S.C. § 289(a)(2) of federal statutes requires NIH to ensure that women are included as subjects in clinical research. Other nations have adopted similar regulations [1]. Despite these statutes and resulting policies, studies have found that discrepancies in study sample sex ratios remain. A survey of 1,800 articles in American medical journals from 1985–1996 found that when only one sex was used to study conditions affecting both sexes, males were used more frequently than females [2]. Another survey of 69 articles in 2004 American medical journals found inadequate compliance with the NIH guidelines [3]. The topic of sex balance in biomedical research designs has gained renewed interest in 2014 when NIH leadership decided to provide additional focus on sex balance in pre-clinical research involving animals and cells [4]. While there is anecdotal evidence that biomechanical studies have often used male subjects when there is no scientifically valid reason to exclude females, there are little hard data to support or refute this perception.

The goals of this study were to assess how often biomechanical studies use study samples that include women and men and test whether that has changed over time. The study was based on a review of abstracts from the 1983, 1993, 2003, and 2013 annual meetings of the American Society of Biomechanics (ASB). Specifically, the aims of this study were to test the hypotheses that three quantities changed from 1983 to 2013: (a) the percentage of abstracts reporting sex of participants, (b) the percentage of abstracts including both male and female participants, and (c) the proportion of abstracts having approximately balanced selected study samples in regards to sex.

## Methods

To assess whether ASB study samples have changed over time, we analyzed 4 years of ASB conference proceedings: 1983 (Rochester, MN), 1993 (Iowa City, IA), 2003 (Toledo, OH), and 2013 (Omaha, NE). We analyzed abstracts for podium and poster presentation at the ASB annual meeting. For an abstract to meet the inclusion criteria, it had to involve humans or animals. Abstracts describing mathematical and computational models were included if they used experimental input data and/or compared model results to experimental data. We examined study sample appropriateness only in regards to sex of participants. We excluded abstracts that applied to genderless or inanimate objects and abstracts involving computational models that collected no empirical data. Furthermore, we excluded studies that report results of *in vitro* cell biology and also any keynote, Borelli Award, and Hay Award lecture abstracts.

The study was designed to analyze 43 randomly selected abstracts from the proceedings of each of the four annual meetings spaced 10 years apart. An exception to this sampling strategy occurred for 1983 in which only 45 abstracts were presented, 36 of which met this study’s inclusion criteria. All eligible 1983 abstracts were selected. We were able to randomly choose 43 abstracts for the 1993, 2003, and 2013 meetings.

We recorded each abstract’s conference year, title, applicable population, and study sample information for abstracts meeting the inclusion criteria. We determined each study’s applicable population by thoroughly reviewing the abstract. Most biomechanics studies apply to both sexes. However, some studies have a sound scientific rationale for excluding one sex. Therefore, we scrutinized the introduction of each abstract to determine if the authors argue that their study is specific to a single sex for a valid scientific reason. Then each abstract was categorized according to whether the research topic applied to (a) both sexes, (b) males only, or (c) females only. Next, each abstract was evaluated to determine if study sample was described with respect to sex. Since study samples were not described in a consistent way, we determined that the study sample was adequately described with respect to sex if the authors (1) explicitly stated the number of females and males studied, (2) stated sexes using abbreviations M and F for male and female, respectively, or (3) stated percentage of participants of one sex (we counted the remaining participants as members of the opposite sex). Sex was also considered to be reported if study participants were recruited from an exclusively male or female organization (for example, a study sample of 20 National Football League linebackers implies male participants and a study on pelvic floor disorders implies female participants). Using these standards we categorized each abstract into (a) both sexes used, (b) males only, (c) females only, or (d) no study sample data reported. If both sexes were included, we recorded the numbers of male and female participants studied. If an abstract contained more than one study sample, we recorded the total number of male and female participants across all samples studied in the same abstract.

We sought to determine if study samples were adequately selected. An abstract was determined to have an “approximately balanced selected study sample” if each sex accounted for 50 ± 20% of study participants or the authors justified a different percentage of male and female participants. Note that abstracts having a valid scientific reason to preclude one sex were categorized as having an approximately balanced selected study sample if they reported including participants of only the applicable sex.

An inter-rater reliability assessment was assessed. Two authors (S.B. and R.E.H.) analyzed a random sample of 40 abstracts from the 2009 ASB meeting (State College, PA).

Descriptive statistics were computed for the percentage of abstracts (a) reporting sex of participants, (b) including both sexes, and (c) being approximately balanced between 1983, 1993, 2003, and 2013. Cohen’s kappa coefficients were also computed for categorical measures, and intra-class correlation coefficients (ICC) were computed for the number of male and female study participants. Logistic regression was used to test for changes over time.

A statistical power calculation was performed to determine the sample size of 43 abstracts per meeting. It was based on an analysis of a random sample of 20 ASB abstracts from the 2012 ASB meeting and an effect size of 0.25.

## Results

Inter-rater agreement was excellent, with kappa coefficients ranging from 0.77 to 1.0 for categorical variables. ICC values for the numbers of female and male study participants were 0.92 and 0.86, respectively.

**Fig 1:**
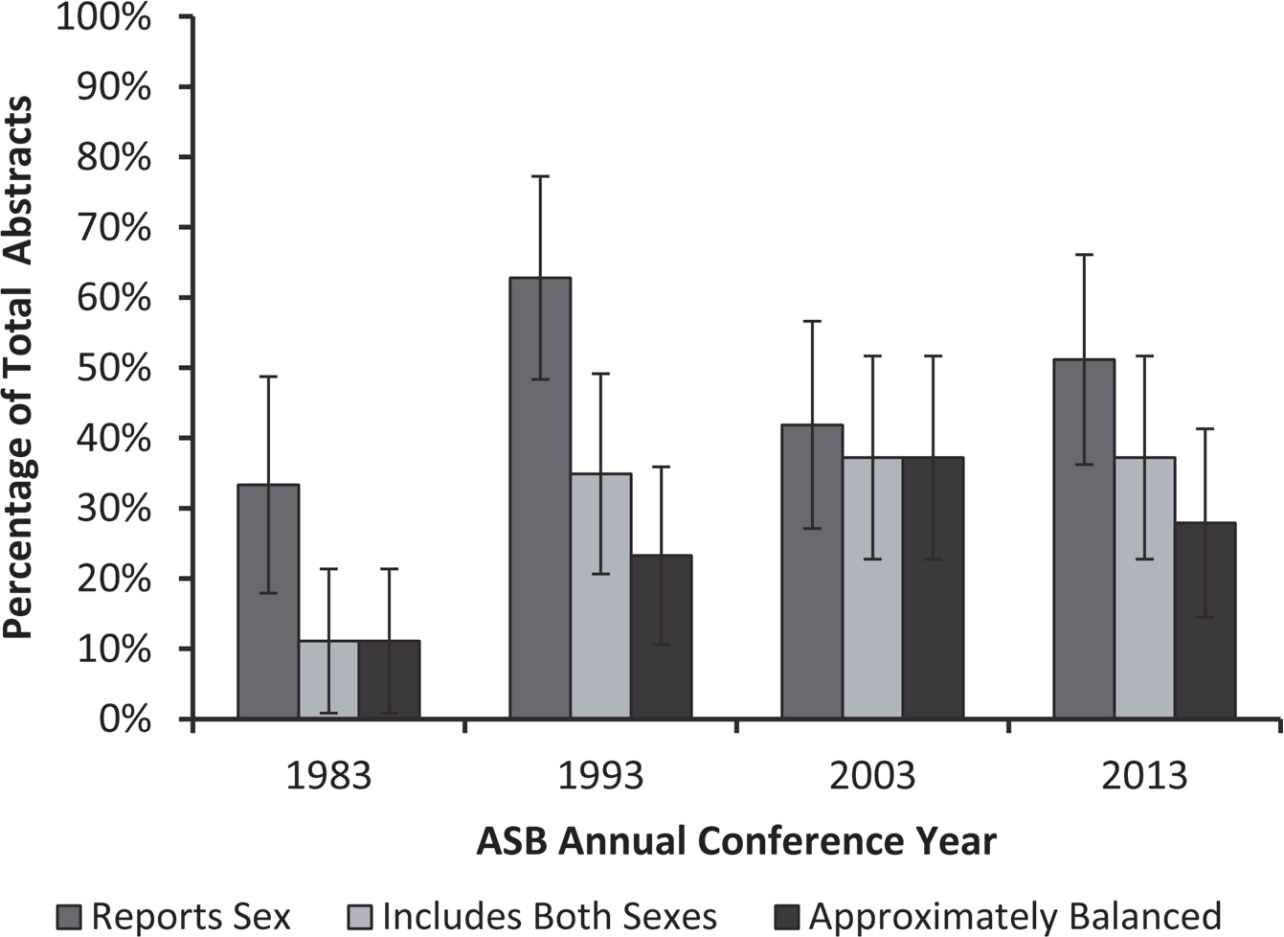
Percentage of American Society of Biomechanics (ASB) conference abstracts that report sex of participants (“Reports Sex”), include both male and female participants (“Includes Both Sexes”), and have study samples that are 50 ± 20% female (“Approximately Balanced”).

The proportion of abstracts reporting sex of study samples ranged from 33% to 63% (Fig. 1). Logistic regression did not reveal a change in this variable across time. The proportion of abstracts reporting results of studies including both sexes ranged from 11% to 37%, and logistic regression indicated a statistically significant increase over time (*p* < 0.05). The proportion of abstracts reporting study samples that were approximately balanced was the lowest of the three measures computed, ranging from 11% to 37%. The proportion of abstracts including both sexes and having approximately balanced study populations achieved their minima in 1983 and maxima in 2003. In both of those years the two measures were equal. Logistic regression also indicated a statistically significant increase in this measure over time (*p* < 0.05).

## Discussion

The objective of this paper was to use abstracts presented at annual meetings of the American Society of Biomechanics to determine how often biomechanical studies use study samples that have an approximate balance between females and males. Secondarily, time trends were also analyzed. The results showed that the highest proportion of abstracts presented during a meeting that had approximately balanced study samples was 37%. This is far less than the ideal of 100%; there appears to be significant room for improvement. However, there has been an encouraging increase over time for the proportions of abstracts including both sexes and having approximately balanced study samples.

This study has several limitations. First, abstracts may not fully represent the actual science being performed due to space limitations. The full papers resulting from research projects may have better reporting of study samples simply because there is more room. A complete assessment of the generalizability of the science being conducted requires evaluating the peer-reviewed publications in addition to conference abstracts. An additional limitation of the paper was the selection of the 50 ± 20% female criteria for a study sample to be approximately balanced. This criteria was selected arbitrarily and the width of the interval was selected to be wide because of the small sample sizes that often occur in biomechanics abstracts. In a small study it is likely that there will be an imbalance of males and females due solely to chance unless a strict ratio is enforced.

## Conclusions

Although an increasing trend was found in the proportion of abstracts including both sexes and being approximately balanced, there is room for continued improvement in both categories as values still remain below 50% of total abstracts. Reporting of sex of study samples is also imperfect, with only 51% of 2013 meeting abstracts including this. We recommend that program chairs consider requiring the reporting of sex in the description of study samples. We hypothesize that the increased transparency resulting from reporting of sex of participants will also encourage authors to think more critically about the subject samples they use.
